# Development of automatic organ segmentation based on positron-emission tomography analysis system using Swin UNETR in breast cancer patients in Korea

**DOI:** 10.12771/emj.2025.00094

**Published:** 2025-04-02

**Authors:** Dong Hyeok Choi, Joonil Hwang, Hai-Jeon Yoon, So Hyun Ahn

**Affiliations:** 1 Department of Medicine, Yonsei University College of Medicine, Seoul, Korea; 2 Medical Physics and Biomedical Engineering Lab, Yonsei University College of Medicine, Seoul, Korea; 3 Department of Radiation Oncology, Yonsei Cancer Center, Heavy Ion Therapy Research Institute, Yonsei University College of Medicine, Seoul, Korea; 4 Department of Nuclear and Quantum Engineering, Korea Advanced Institute of Science and Technology, Daejeon, Korea; 5 Medical Image and Radiotherapy Lab, Korea Advanced Institute of Science and Technology, Daejeon, Korea; 6 Department of Nuclear Medicine, Ewha Womans University School of Medicine, Seoul, Korea; 7 Department of Biomedical Engineering, Ewha Womans University College of Medicine, Seoul, Korea; 8 Ewha Medical Research Institute, Ewha Womans University College of Medicine, Seoul, Korea; 9 Ewha Medical Artificial Intelligence Research Institute, Ewha Womans University College of Medicine, Seoul, Korea

**Keywords:** Artificial intelligence, Breast neoplasms, Deep learning, Positron emission tomography, Prognosis, Republic of Korea

## Abstract

**Purpose:**

The standardized uptake value (SUV) is a key quantitative index in nuclear medicine imaging; however, variations in region‐of‐interest (ROI) determination exist across institutions. This study aims to standardize SUV evaluation by introducing a deep learning‐based quantitative analysis method that enhances diagnostic and prognostic accuracy.

**Methods:**

We used the Swin UNETR model to automatically segment key organs (breast, liver, spleen, and bone marrow) critical for breast cancer prognosis. Tumor segmentation was performed iteratively based on predefined SUV thresholds, and prognostic information was extracted from the liver, spleen, and bone marrow (reticuloendothelial system). The artificial intelligence training process employed 3 datasets: a test dataset (40 patients), a validation dataset (10 patients), and an independent test dataset (10 patients). To validate our approach, we compared the SUV values obtained using our method with those produced by commercial software.

**Results:**

In a dataset of 10 patients, our method achieved an auto‐segmentation accuracy of 0.9311 for all target organs. Comparison of maximum SUV and mean SUV values from our automated segmentation with those from traditional single‐ROI methods revealed differences of 0.19 and 0.16, respectively, demonstrating improved reliability and accuracy in whole‐organ SUV analysis.

**Conclusion:**

This study successfully standardized SUV calculation in nuclear medicine imaging through deep learning‐based automated organ segmentation and SUV analysis, significantly enhancing accuracy in predicting breast cancer prognosis.

## Introduction

### Background

Breast cancer (BC) is the most common cancer among women and one of the leading causes of cancer‐related deaths worldwide [1,2]. With recurrence rates of 20%–30%, accurate prognostic prediction is essential for treatment planning [3]. Key prognostic factors include tumor size, nuclear grade, axillary lymph node involvement, hormone receptor status, and the Ki‐67 proliferation index [4].

18F fluorodeoxyglucose positron-emission tomography/computed tomography (18F-FDG PET/CT) is widely used to evaluate tumor metabolism, stage cancer, and guide treatment decisions [5]. PET-derived parameters such as standardized uptake values (SUVs), metabolic tumor volume, and total lesion glycolysis (TLG) have emerged as significant prognostic indicators in BC [6,7].

Traditional segmentation methods such as region-growing, thresholding, and level-set techniques have been widely used, yet they require manual intervention and suffer from interobserver variability. The Swin UNETR model, which integrates Swin transformers with the UNETR architecture, offers improved spatial feature representation and enhanced segmentation accuracy. However, its computational cost and real-world feasibility remain areas of concern, as discussed later in this study.

Systemic inflammatory responses also influence cancer progression and prognosis [8]. Biomarkers such as the neutrophil-to-lymphocyte ratio and platelet-to-lymphocyte ratio have been associated with outcomes in multiple cancers [9]. The reticuloendothelial system (RES), which includes the bone marrow, spleen, and liver, plays a key role in systemic inflammation. FDG PET has been used to evaluate metabolic activity in these organs, and increased RES activity has been linked to poor prognosis in BC and other cancers [10,11].

Accurate delineation of BC lesions and RES organs is essential for quantifying PET parameters. Although manual segmentation by nuclear medicine physicians is the current gold standard, it is time-consuming and prone to interobserver variability, which can lead to potential errors [12]. Automated segmentation methods are therefore necessary to reduce variability and improve efficiency.

### Objectives

**Fig. 1.**
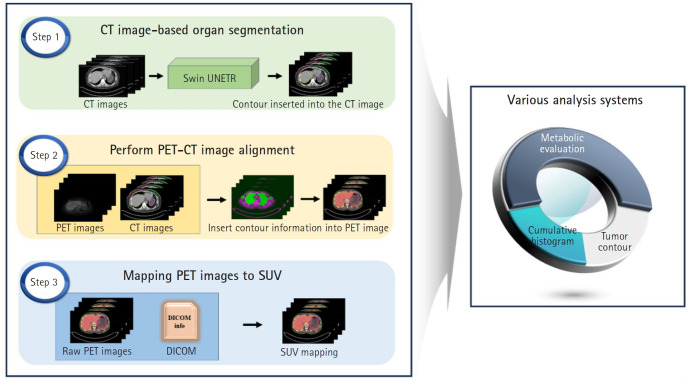
Schematic representation of the study flow. The left panel summarizes the steps involved in the study, while the right panel illustrates the inherent functionalities of the research findings. CT, computed tomography; PET, positron emission tomography; SUV, standardized uptake value.

This study employs the Swin-UNETR architecture to segment both the breast and RES organs, highlighting their prognostic significance. Our goal is to develop an advanced diagnostic and prognostic system for BC. We aimed to automate the identification of tumor location and size using SUVs derived from PET/CT scans of BC patients. In addition, we developed a computational tool to calculate SUVs in organs associated with prognosis. A schematic representation of the study flow is shown in Fig. 1.

## Methods

### Ethics statement

All patient data used in this research were reviewed and approved by the Institutional Review Board (IRB) of Ewha Womans University Mokdong Hospital (IRB no., 2023-07-001-002). Obtaining informed consent from individual patients was exempt because of the retrospective design of this study.

### Study design

This study is a retrospective, medical record-based prediction study. It is reported in accordance with the TRIPOD-artificial intelligence (AI) reporting guidelines for articles on deep learning in the medical field (development or prediction), available at https://www.tripod-statement.org/.

### Setting

Data were collected between 2012 and 2014 from female patients (mean age, 54.5±10.3 years; range, 33–77 years) who underwent FDG-PET/CT for initial BC staging at Ewha Womans University Mokdong Hospital in Korea.

### Participants

A total of 60 patients were included in the study. All eligible patients diagnosed during the study period were included. Among them, 32 underwent mastectomy, while 28 received breast-conserving surgery.

### Data source

PET/CT scans were performed using a Siemens Biograph mCT system (128-slice CT; Siemens Medical Solutions). Patients fasted for at least 6 hours before scanning, and FDG was administered when blood glucose levels were below 140 mg/dL. PET/CT images were acquired 60 minutes after an intravenous FDG injection (5.18 MBq/kg), covering the skull base to mid-thigh. First, non-contrast CT images were obtained (120 kVp, 50 mAs, 1.2 pitch), followed by 3D PET image acquisition (2 minutes per bed position, covering 5 to 7 positions). PET images were reconstructed using a 3D-OSEM iterative algorithm (2 iterations, 21 subsets) with time-of-flight and point-spread function corrections.

### Outcome variables

The outcome variables included segmentation results, PET/CT alignment, SUVs, and TLG.

### Study size

No sample size estimation was performed, as all eligible subjects were included in the study.

### Deep learning models

#### Auto segmentation

**Fig. 2.**
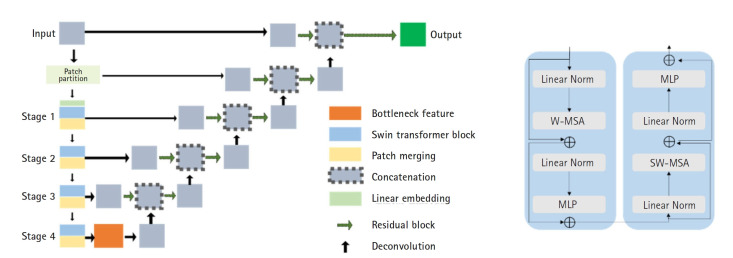
The architecture (A) and transformer blocks (B) of Swin UNETR. MLP, multilayer perceptron; W-MSA, window-based multi-head self-attention; SW-MSA, shifted window-based self-attention.

For AI model training, manual segmentation of CT scans from 60 patients was performed using MIM software (MIM Software Inc.), focusing on the breasts, liver, spleen, and bone marrow. An experienced physician performed the segmentation, which was then verified by a second experienced physician. In cases of discrepancy, the 2 physicians reached a consensus. The data were divided into a training set (40 patients), a validation set (10 patients), and an independent test set (10 patients). The Swin UNETR model, a deep learning architecture that integrates the Swin Transformer with convolutional neural networks, was employed for segmentation (Fig. 2).

Encoder: CT images were divided into non-overlapping 2×2×2 patches, with each patch represented as a 48-dimensional feature vector. These patches were embedded into a sequence representation and tokenized at a resolution of (H/2×W/2×D/2). The encoded features were processed through 2 consecutive Swin Transformer blocks that utilized window-based multi-head self-attention (W-MSA) and shifted window-based self-attention (SW-MSA) (Fig. 2B). A patch-merging layer then reduced the number of tokens while doubling the feature dimensions, and this process was repeated 4 times throughout the encoding stage.

Decoder: The encoded features were reshaped to a resolution of (H/32×W/32×D/32) and processed through residual blocks comprising 3×3×3 convolutional layers with instance normalization. These features were then up-sampled using deconvolutional layers and concatenated with outputs from previous stages. The final encoder output was integrated with these processed features and passed through another residual block. A final 1×1×1 convolutional layer with a SoftMax activation function produced segmentation probabilities.

Model parameters: Various preprocessing techniques were employed to enhance segmentation accuracy. Pixel values were normalized between 0 and 1 by clipping those outside the range of –175 to 250. CT image resolution was standardized at 1.5×1.5×2.0 mm^3^, and images were randomly cropped to 96×96×96. Data augmentation techniques included random flipping and rotation (with a probability of 0.1) along all 3 axes, and intensity shifting (with a probability of 0.5 and an offset of 0.1). The model was trained using a combination of dice loss and cross-entropy loss, optimized with the Adam optimizer and stabilized using GradScaler (PyTorch). Training was conducted over 20,000 iterations.

#### SUV acquisition

PET/CT alignment: To transfer CT-based segmentation to PET images, multimodal image alignment was performed using MATLAB’s alignment module. The optimizer was configured with an initial radius of 0.009, an epsilon of 1.5E-4, and a maximum of 1,000 iterations for optimal performance. Segmentation accuracy was evaluated by comparing contour coordinates from CT-based segmentation using the dice coefficient. PET contours were aligned to CT contours using MIM software, and the PET/CT alignment metric was used to assess accuracy. This analysis was conducted on 2 patients, with image details provided in Table 1.

Convert pixel values to SUVs of PET: SUVs were calculated using patient-specific parameters extracted from DICOM data (https://www.dicomstandard.org/), including acquisition time, radiopharmaceutical start time, radionuclide half-life, total dose, rescale slope, and patient weight. These factors enabled accurate SUV quantification per voxel, facilitating metabolic activity assessment for each organ. SUVs were body weight-based and computed using the following formula:


(1) $${\text{SUV}}_{body\;weight\left(\frac{kg}{cc}\right)}=\frac{\left(pixel\;value\;\times Dicom\;rescale\;factor\;\times Patient\;weight\right)}{Total\;dose\times \;e^{\left(\frac{-\text{log}\left(2\right)\times \left(Series\;time\;-\;Radiophamaceutical\;start\;time\right)\;}{F^{18}-FDG\;half\;life\;time}\right)}}$$

where pixel value represents the PET image’s raw intensity, and the DICOM rescale factor normalizes the pixel arrays. “Series time” refers to the scan initiation time, while “radiopharmaceutical start time” marks the time of 18F FDG administration.

### Evaluation metrics

#### Auto segmentation

The Swin-UNETR model was employed to segment the breasts, spleen, liver, and bone marrow. Evaluation was conducted qualitatively by comparing predicted CT images with manually labeled organ structures, and quantitatively by calculating the average dice coefficient and loss over 20,000 iterations.

#### Organ-level SUV evaluation

To assess the accuracy of SUV measurements, maximum SUV (SUVmax) and mean SUV (SUVmean) values were compared using different PET image analysis methods. SUVmax was extracted from each contoured organ and compared with values obtained via MIM software, ensuring that the volume of interest (VOI) excluded adjacent organs. SUVmean was evaluated using: (1) a single VOI in MIM software, (2) a single VOI in our program, and (3) A whole-organ contour in our program.

Both SUVmax and SUVmean were consistently derived across methods using a fixed 1.2 cm radius VOI centered at each organ’s centroid. Statistical analysis was performed to characterize the results.

#### Tumor contour based on SUV

Organ contour labels were aligned with PET images to localize tumors. By using registered contour labels and SUV maps, tumors were identified based on threshold values of 40% and 50% of SUVmax. This approach provided tumor coordinate information and enabled visualization of tumor size and location on CT images.

#### Total lesion glycolysis evaluation

TLG quantifies metabolic activity by integrating tumor size and SUV. It is calculated by multiplying each lesion’s SUV by its corresponding volume and summing these values across all lesions within a given region of interest. In this study, TLG was measured using SUV thresholds of 40% and 50%.

#### Cumulative SUV histogram

The cumulative SUV histogram represents intertumoral heterogeneity by plotting the percentage of tumor volume that exceeds specified SUV thresholds. This method provides a concise summary of tumor metabolic characteristics. Cumulative SUV histograms were generated for all segmented organs in PET images.

Python code for this study is available at Supplement 1.

### Statistical methods

Descriptive statistics were calculated.

## Results

### Auto segmentation

**Fig. 3.**
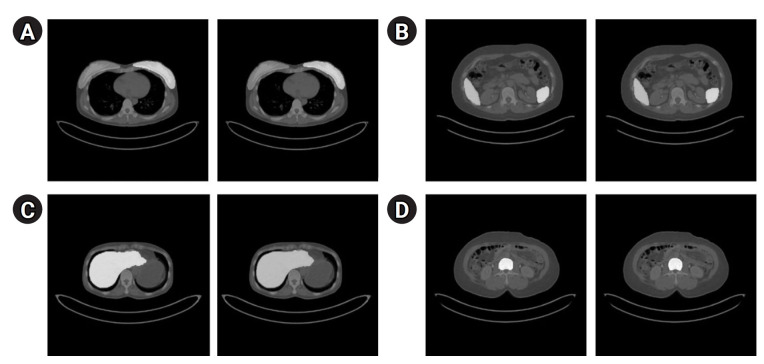
Segmentation results of the Swin-UNETR model: breast (A), spleen (B), liver (C), and bone marrow (D). In each case, the left image shows the label, and the right image shows the predicted result.

**Fig. 4.**
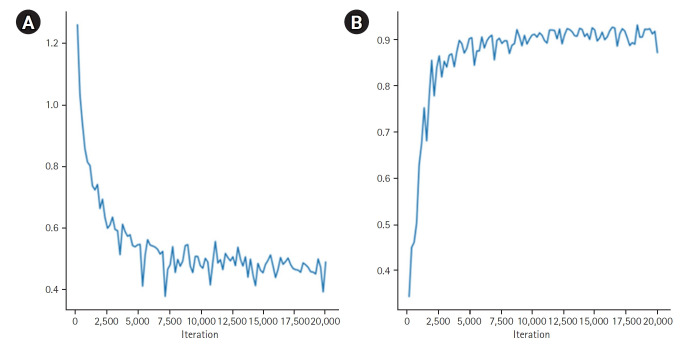
Quantitative results of organ segmentation using Swin-UNETR: loss (A) and dice scores (B) during 20,000 iterations.

Fig. 3 presents the segmentation results for the breast, spleen, liver, and bone marrow using the Swin-UNETR model. The left images display the organ labels, and the right images show the predicted segmentation. Fig. 4 provides quantitative results, revealing a maximum dice coefficient of 0.9311 and a minimum loss of 0.3813, which demonstrates the model’s effectiveness in accurately segmenting organs with diverse shapes and sizes. These results were obtained from the validation dataset during the training process.

### PET/CT alignment

**Fig. 5.**
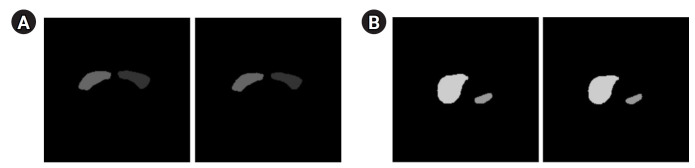
Results of positron emission tomography/computed tomography alignment using the MIM program for breast (A), and liver and spleen (B). In each image, the left side displays results from the MIM program, while the right side shows results from our technique.

A comparison of the dice scores for the CT-based contours generated by the MIM program vs. our alignment program, using datasets from 2 patients, yielded dice coefficients of 0.9114 and 0.9315, respectively (Fig. 5).

### SUV acquisition

SUVmax comparisons: Table 2 summarizes the SUVmax comparisons between the MIM software and our method, showing an average difference of 0.23 (range, 0.11–0.35). The liver exhibited the highest difference (0.35) due to its larger volume, whereas the spleen showed the lowest difference (0.11).

SUVmean comparisons: Table 3 presents the SUVmean comparisons. When using a single VOI, the average difference between our program and the MIM software was 0.138 (range, 0.07–0.24), with the highest variation observed in the liver (0.24) and the lowest in the right breast (0.07). When using organ contours, the difference increased to an average of 0.27 (range, 0.26–0.30), with the greatest variation in the spleen (0.30). The differences between the single VOI and organ contour methods were consistently larger than those observed between the 2 single VOI methods, highlighting the impact of VOI selection.

### SUV-based tumor contour

**Fig. 6.**
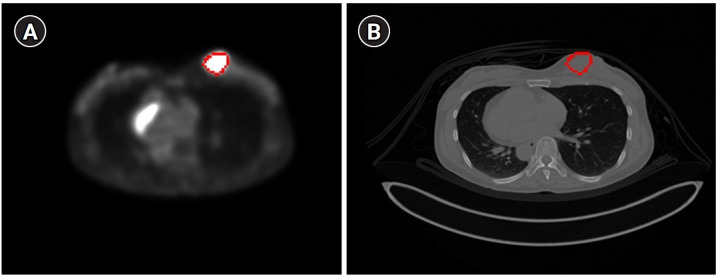
The tumor with a maximum standardized uptake value greater than the 50% threshold displayed on a breast cancer (BC) positron emission tomography image (A) and a BC computed tomography image (B) using the contour method.

Fig. 6 displays the results of contour-based insertion of volumetric information, specifically regions with SUVmax exceeding the 50% threshold based on BC patients’ PET images. This approach enables the identification of tumor location and size on both PET and CT images.

### TLG evaluation

Table 4 presents the TLG values obtained for 10 patients, focusing on the breast region where the tumors were located.

### Cumulative SUV histogram

**Fig. 7.**
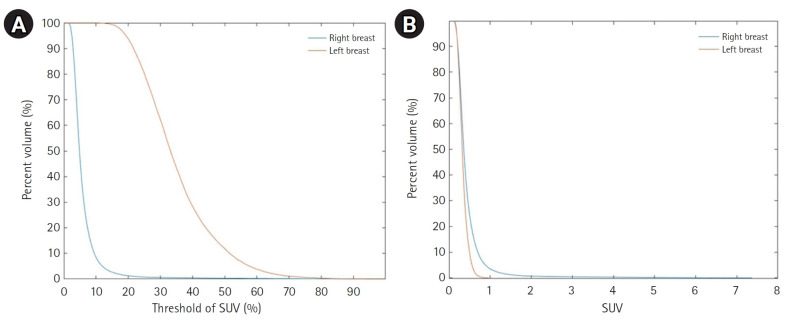
Cumulative standardized uptake value (SUV) histogram results for a breast cancer patient with a tumor in the right breast. (A) The x-axis represents the SUV threshold (%) and (B) the x-axis represents absolute SUV values.

The cumulative SUV histogram was used to analyze the proportion of organ volume exceeding specific SUV thresholds. Fig. 7 shows cumulative SUV histograms for a BC patient with a right breast tumor and no lesions in the left breast. The tumor histogram exhibits a convex downward shape, indicating a higher proportion of tumor volume with SUV values above the threshold. In contrast, the healthy breast histogram shows a convex upward shape, suggesting a lower proportion of high-SUV regions.

## Discussion

### Key results

This study introduces a novel automated method for PET image segmentation and quantitative evaluation based on the Swin UNETR architecture. The Swin-UNETR model achieved a dice coefficient of 0.9311 and a loss of 0.3813 for precise organ segmentation. PET/CT alignment produced dice scores of 0.9114 and 0.9315. Comparisons of SUVmax values revealed an average difference of 0.23, while SUVmean differences were 0.138 when using a single VOI and 0.27 when using organ contours. Tumor contouring successfully identified regions where SUVmax exceeded 50%. Additionally, TLG values effectively quantified tumor metabolic activity, and cumulative SUV histograms distinguished tumor tissue from healthy tissue through distinct patterns.

### Interpretation/comparison with previous studies

Previous methods—including thresholding, gradient-based techniques, and region growing—suffer from limitations such as manual parameter adjustments, sensitivity to noise, and difficulty in handling complex structures [13-15]. Our approach improves segmentation accuracy by leveraging automated CT-based segmentation applied to PET images, which allows for precise organ delineation and accurate SUV extraction. By converting PET pixel values to SUVs using DICOM data, our method ensures reproducibility in SUV measurements [16,17]. In contrast to conventional clinical approaches that rely on variable region-of-interest selection, our method standardizes the calculation of SUVmax and SUVmean for specific organs, thereby enhancing reliability. Moreover, analyzing SUVs from both tumor and normal tissues offers valuable predictions for postoperative outcomes, given that systemic inflammatory responses are key prognostic indicators in cancer. 18F FDG PET/CT is widely used for assessing tumor metabolism and systemic inflammation, especially in organs such as the spleen, liver, and bone marrow, which are pivotal in cancer progression.

Beyond SUV analysis, our method incorporates metabolic tumor volume and TLG, both of which are crucial for evaluating tumor burden and treatment response. By integrating SUV and tumor volume, TLG offers insights into tumor aggressiveness; if used in radiation therapy planning, this approach may lead to more effective treatment strategies.

A comparative analysis revealed differences in SUVmean values between our method and commercially available MIM software. Because MIM calculates SUVmean using a circular VOI that does not fully capture organ shape, slight variations occur. This finding emphasizes the importance of precise VOI selection and contouring techniques when evaluating SUVs, especially for SUVmean calculations.

Higher TLG values indicate increased glycolytic activity, suggesting greater tumor aggressiveness, and underscore TLG’s value as a biomarker for BC tumor burden and metabolic characteristics. By quantifying metabolic activity, TLG provides valuable information for treatment planning and monitoring.

Although TLG findings are clinically significant, our current study does not include follow-up data on tumor recurrence or patient mortality. Future research will evaluate the correlation between TLG and clinical outcomes by incorporating both short-term data for recurrence assessment and long-term data for survival analysis, thereby providing deeper insights into the prognostic value of TLG in BC management.

### Limitations

Inter-observer agreement could not be assessed because segmentation was performed by a single physician and subsequently verified by another, rather than being conducted independently by multiple annotators. This approach may introduce potential bias in the segmentation process. Future studies that include multiple independent annotations and calculate agreement metrics could further validate the robustness of this segmentation methodology.

### Clinical implications

Our method offers several clinical advantages. Automated PET segmentation facilitates early cancer detection and diagnosis while improving treatment planning. Quantitative metabolic lesion evaluation using SUVs and TLG functions as an independent prognostic factor that improves patient stratification and monitoring. Additionally, our approach reduces inter-observer variability, streamlines workflow, and enhances the efficiency of image interpretation, ultimately leading to better patient management.

Although the training phase of the Swin UNETR model requires substantial computational resources, the inference process—where the trained model segments new scans—is highly efficient and can be completed within seconds per scan. If further validated, this approach could be integrated into clinical programs to support automated segmentation. Moreover, applying optimization techniques such as model pruning and mixed-precision inference could further enhance its real-time applicability in clinical workflows. While this study focuses on the feasibility of using Swin UNETR for segmentation and SUV quantification, the impact of variations in SUV calculation methods on clinical decision-making remains to be fully understood. Future research should investigate how these variations influence prognosis prediction and treatment response assessment, thereby confirming its practical utility in clinical workflows.

### Suggestion for further studies

Despite the demonstrated benefits, challenges persist in cases with a low signal-to-noise ratio or atypical PET images. Further optimization is required to minimize errors during the transfer of CT-based contours to PET images. Additionally, we plan to extend the segmentation to include the lungs and lymph nodes—common metastatic sites in BC—to improve metastasis detection and prognosis prediction. Future research will concentrate on refining the algorithm’s performance and expanding its capabilities to manage more complex cases.

### Conclusion

This study introduces an automated PET segmentation and evaluation method that enhances diagnostic accuracy and supports treatment planning in BC patients. Further optimization is needed to address remaining segmentation challenges and to broaden its clinical applications.

## Figures and Tables

**Table 1.** Image size and slice thickness of the PET/CT images from 2 patients were used to verify PET/CT alignment

	Patient 1		Patient 2	
	CT	PET	CT	PET
Image size (pixels)	512×512×284	200×200×283	512×512×462	200×200×284
Slice thickness (mm)	3	3	2	3

CT, computed tomography; PET, positron emission tomography.

**Table 2.** Comparison of the SUVmax results obtained from the MIM software and our methodology

Patient index	Methods for obtaining SUV_max_	Target organs				
		Breast_R	Breast_L	Liver	Spleen	Bone marrow
1	MIM program result	12.03	1.59	2.51	1.68	1.50
	Program results contour	11.97	1.65	2.48	1.56	1.65
2	MIM program result	4.67	0.66	3.10	1.43	1.13
	Program results contour	4.31	0.68	3.66	1.25	1.23
3	MIM program result	10.96	1.96	5.43	4.47	2.60
	Program results contour	10.79	2.18	5.98	4.71	2.74
4	MIM program result	7.03	1.14	6.12	2.15	2.46
	Program results contour	6.92	1.36	6.44	1.95	2.63
5	MIM program result	1.05	7.38	2.87	2.46	2.32
	Program results contour	0.96	7.37	3.06	2.51	2.68
6	MIM program result	7.89	1.99	2.92	2.05	2.70
	Program results contour	7.15	1.77	2.06	2.00	2.61
7	MIM program result	6.91	2.54	2.38	2.14	2.29
	Program results contour	7.55	2.77	2.18	2.10	2.18
8	MIM program result	5.29	1.50	3.09	2.20	3.90
	Program results contour	5.45	1.60	3.12	2.18	4.14
9	MIM program result	4.68	2.58	6.60	3.69	3.19
	Program results contour	5.47	2.68	6.99	3.68	3.56
10	MIM program result	1.58	3.61	4.54	1.93	2.10
	Program results contour	1.83	3.32	4.19	1.73	2.30
Average difference	MIM program result-Program results contour	0.34±0.27	0.15±0.09	0.35±0.25	0.11±0.08	0.19±0.10

Values are presented as number or mean±standard deviation. Each organ was analyzed in 10 patients. SUVmax, maximum standardized uptake value.

**Table 3.** SUVmean results for each of the organs analyzed in 10 patients

Patient index	Methods for obtaining SUV_mean_	Target organs				
		Breast (right)	Breast (left)	Liver	Spleen	Bone marrow
1	MIM program result	0.76	0.74	2.18	1.30	1.23
	Program results in one VOI	0.84	0.71	1.96	1.30	1.21
	Program results contour	1.14	0.81	1.73	1.16	0.98
2	MIM program result	0.57	0.32	1.14	0.98	0.45
	Program results in one VOI	0.49	0.27	0.98	0.89	0.44
	Program results contour	0.47	0.30	1.11	0.77	0.61
3	MIM program result	1.24	1.53	1.80	2.15	1.13
	Program results in one VOI	1.28	1.40	1.85	2.02	1.17
	Program results contour	0.60	0.43	2.34	1.38	1.10
4	MIM program result	0.64	0.45	2.06	1.97	2.08
	Program results in one VOI	0.54	0.51	1.69	1.71	1.72
	Program results contour	0.59	0.45	1.67	1.48	1.37
5	MIM program result	0.55	0.45	2.19	2.12	1.32
	Program results in one VOI	0.36	0.41	1.79	1.90	1.07
	Program results contour	0.44	0.34	2.02	1.62	1.19
6	MIM program result	0.94	0.76	2.23	1.59	1.67
	Program results in one VOI	0.86	0.86	1.92	1.56	1.40
	Program results contour	0.59	0.40	1.94	1.39	1.39
7	MIM program result	1.09	0.96	1.56	1.49	0.90
	Program results in one VOI	1.13	1.58	1.80	1.66	0.80
	Program results contour	0.69	0.65	1.83	1.40	1.32
8	MIM program result	0.88	0.54	2.44	1.90	0.70
	Program results in one VOI	0.89	0.53	2.23	1.70	0.60
	Program results contour	0.50	0.38	2.13	1.45	1.34
9	MIM program result	0.32	0.41	2.42	1.90	1.77
	Program results in one VOI	0.39	0.44	2.24	2.00	1.76
	Program results contour	0.40	0.35	2.30	1.84	1.68
10	MIM program result	1.02	1.07	1.38	0.52	0.73
	Program results in one VOI	1.02	1.07	1.09	0.44	0.94
	Program results contour	0.58	0.68	1.32	0.58	0.75
Average difference	MIM program result; program results in one VOI	0.07±0.05	0.11±0.18	0.24±0.10	0.13±0.08	0.14±0.12
	MIM program result; program results contour	0.29±0.19	0.26±0.31	0.26±0.16	0.30±0.23	0.27±0.23

Values are presented as number or mean±standard deviation. A comparison of the SUVmean results obtained using the MIM program and our methodology using a single VOI, with the SUVmean obtained from the contoured organs shown. SUVmean, mean standardized uptake value; VOI, volume of interest.

**Table 4.** Tumor locations and their TLG values in the 10 patients

Patient index	Breast cancer location	TLG	
		Threshold (40%)	Threshold (50%)
1	Right breast cancer in the inner pericentral area	5,794	4,551
2	Right breast cancer in the upper pericentral area	1,714	1,382
3	Right breast cancer in the upper outer area	1,185	889
4	Right breast cancer in the lower center area	1,898	1,296
5	Left breast cancer in the upper outer area	2,052	1,004
6	Right breast cancer in the lower pericentral area	3,098	1,893
7	Right breast cancer in the lower pericentral area	3,513	2,586
8	Right breast cancer in the upper outer area	1,358	733
9	Metastatic lymph node in the right axilla	383	275
10	Left breast cancer in the upper outer area	2,909	2,216

TLG was calculated by setting the maximum SUV threshold at 40% and 50% and calculating the mean SUV. TLG, total lesion glycolysis; SUV, standardized uptake value.

## Supplementary materials

Supplement 1. Python code used in this study is available at https://github.com/DonghyeokChoi/breast_pet.git.
